# Crystal structure of poly[[*trans*-di­aqua­bis­[μ_2_-*trans*-4,4′-(diazenedi­yl)dipyridine]­nickel(II)] diiodide ethanol disolvate]

**DOI:** 10.1107/S1600536814016158

**Published:** 2014-08-01

**Authors:** Josefina Perles, Miguel Cortijo, Santiago Herrero

**Affiliations:** a Departamento de Química Inorgánica I, Facultad de Ciencias Químicas, Universidad Complutense de Madrid, Spain

**Keywords:** crystal structure, nickel coordination compound, bidimensional MOF, cationic network

## Abstract

In the title compound, {[Ni(C_10_H_8_N_4_)_2_(H_2_O)_2_]I_2_·2C_2_H_5_OH}_*n*_, the complex shows an octa­hedral environment of the Ni^2+^ cation in which it is located on a centre of symmetry, linked to two water mol­ecules and the pyridine-N atoms of four 4,4′-(diazenediyl)dipyridine ligands bridging Ni^2+^ cations along the *b*- and *c*-axis directions, giving rise to a two-dimensional arrangement. The Ni—N bond lengths are in the range 2.109 (4)–2.186 (3) Å and the Ni—O bond length is 2.080 (3) Å. The 4,4′-(diazenedi­yl)dipyridine ligand lies on an inversion centre. An O—H⋯O hydrogen-bond inter­action is observed between water and ethanol mol­ecules. The I^−^ ions can be regarded as free anions in the crystal lattice.

## Related literature   

For related two-dimensional structures, see: Carlucci *et al.* (2003 ▶); Noro *et al.* (2005 ▶, 2006 ▶); Li *et al.* (2007 ▶); Pan *et al.* (2010 ▶); Aijaz *et al.* (2011 ▶). 
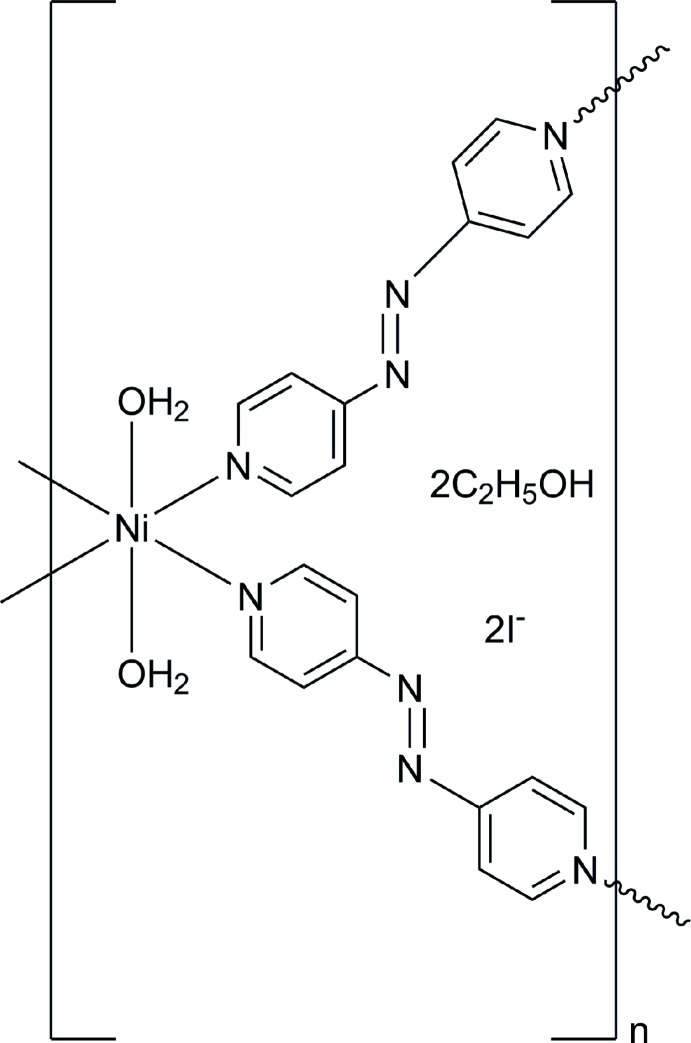


## Experimental   

### Crystal data  

[Ni(C_10_H_8_N_4_)_2_(H_2_O)_2_]I_2_·2C_2_H_6_O
*M* *_r_* = 809.09
Monoclinic, 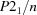

*a* = 8.6367 (11) Å
*b* = 13.2598 (16) Å
*c* = 13.4188 (14) Å
β = 101.737 (3)°
*V* = 1504.6 (3) Å^3^
*Z* = 2
Mo *K*α radiation
μ = 2.74 mm^−1^
*T* = 100 K
0.12 × 0.08 × 0.06 mm

### Data collection  

Bruker Kappa APEXII diffractometer
Absorption correction: multi-scan (*SADABS*; Bruker, 2009 ▶) *T*_min_ = 0.77, *T*_max_ = 0.85
19224 measured reflections
2741 independent reflections
1948 reflections with *I* > 2σ(*I*)
*R*_int_ = 0.067

### Refinement  

*R*[*F* ^2^ > 2σ(*F* ^2^)] = 0.041
*wR*(*F* ^2^) = 0.097
*S* = 1.00
2741 reflections
185 parameters
3 restraints
H atoms treated by a mixture of independent and constrained refinement
Δρ_max_ = 0.95 e Å^−3^
Δρ_min_ = −0.86 e Å^−3^

### 

Data collection: *APEX2* (Bruker, 2009 ▶); cell refinement: *SAINT* (Bruker, 2009 ▶); data reduction: *SAINT*; program(s) used to solve structure: *SHELXS97* (Sheldrick, 2008 ▶); program(s) used to refine structure: *SHELXL97* (Sheldrick, 2008 ▶); molecular graphics: *SHELXTL* (Sheldrick, 2008 ▶); software used to prepare material for publication: *SHELXL97*.

## Supplementary Material

Crystal structure: contains datablock(s) global, I. DOI: 10.1107/S1600536814016158/bx2463sup1.cif

Structure factors: contains datablock(s) I. DOI: 10.1107/S1600536814016158/bx2463Isup2.hkl

Supporting information file. DOI: 10.1107/S1600536814016158/bx2463Isup3.cdx

Supporting information file. DOI: 10.1107/S1600536814016158/bx2463Isup4.docx

**Fig. 1.**
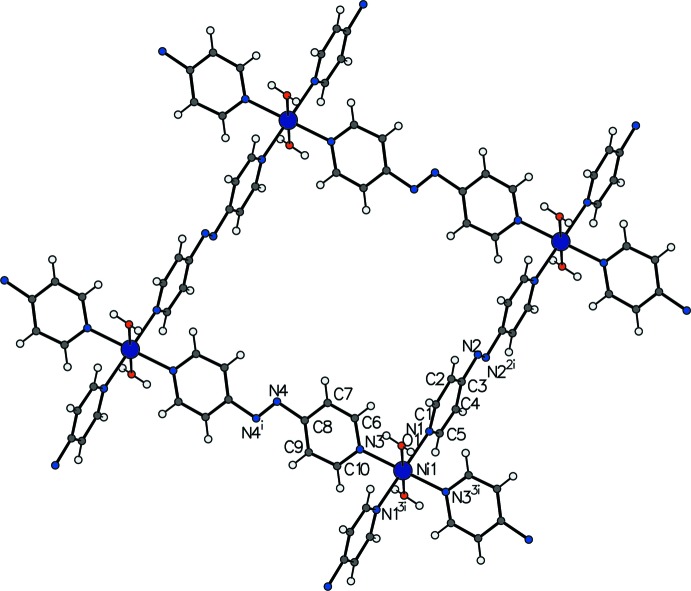
Part of the polymeric structure for the title compound. Symmetry code for compound (i):-x, -y+2, -z+2; (2i): -x-y+1,-z+1;(3i):-x,-y+1,-z+2.

. DOI: 10.1107/S1600536814016158/bx2463fig1.tif
Part of the polymeric structure for the title compound. Symmetry code for compound (i):-x, −y+2, −z+2; (2i): −x-y+1,-z+1;(3i):-x,-y+1,-z+2.

**Fig. 2.**
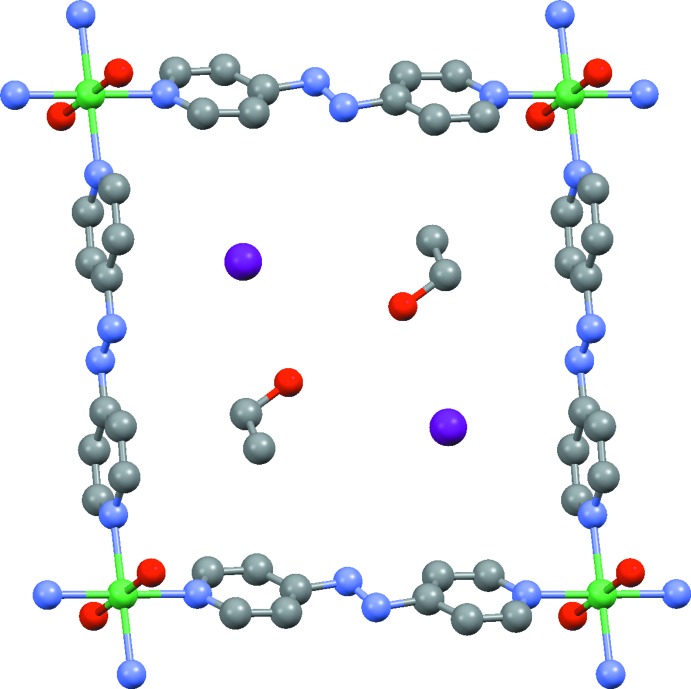
Simplified drawing of a layer parallel to (011). Hydrogen atoms have been omitted for clarity.

. DOI: 10.1107/S1600536814016158/bx2463fig2.tif
Simplified drawing of a layer parallel to (011). Hydrogen atoms have been omitted for clarity.

CCDC reference: 1013422

Additional supporting information:  crystallographic information; 3D view; checkCIF report

## Figures and Tables

**Table 1:** Hydrogen-bond geometry (Å, °)

*D*—H⋯*A*	*D*—H	H⋯*A*	*D*⋯*A*	*D*—H⋯*A*
O1—H1*A*⋯O2^i^	0.83 (4)	1.91 (4)	2.703 (6)	161 (5)

Symmetry code: (i) 
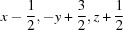
.

## supplementary crystallographic information

### S1. Related Literature

A similar laminar structure was found for the compound [Ni(NCS)_2_(t-apy)_2_]·3toluene (Noro *et al.*, 2006) although in this latter case there is no one-dimensional H-bond chain. For related 2D structures see: Carlucci *et al.* (2003); Noro *et al.* (2005 and 2006); Li *et al.* (2007); Pan *et al.* (2010); and Aijaz *et al.* (2011).

### S2. Preparation

Nickel(II) iodide (0.30 g, 1.0 mmol), *trans*-4,4'-(diazenediyl)di­pyridine (0.18 g, 1.0 mmol), ethanol (9 mL), and water (3 mL) were placed into an 85 mL Teflon vessel with a magnetic stirrer. The vessel was sealed with a lid equipped with a temperature sensor and placed in a ETHOS ONE microwave oven. The reaction mixture was heated for 3 hours at 120°C and left to cool afterwards. Slow inter­diffusion of di­ethyl ether in the obtained solution gave rise to red crystals suitable for single-crystal X-ray diffraction after a few days.

### S3. Refinement

Crystal data, data collection and structure refinement details are summarized in Table 1.

### Crystal data

**Table:**

[Ni(C_10_H_8_N_4_)_2_(H_2_O)_2_]I_2_·2C_2_H_6_O	*F*(000) = 796
*M**_r_* = 809.09	*D*_x_ = 1.786 Mg m^−^^3^
Monoclinic, *P*2_1_/*n*	Mo *K*α radiation, λ = 0.71073 Å
*a* = 8.6367 (11) Å	Cell parameters from 3456 reflections
*b* = 13.2598 (16) Å	θ = 2.9–21.6°
*c* = 13.4188 (14) Å	µ = 2.74 mm^−^^1^
β = 101.737 (3)°	*T* = 100 K
*V* = 1504.6 (3) Å^3^	Prismatic, clear orange–red
*Z* = 2	0.12 × 0.08 × 0.06 mm

### Data collection

**Table:**

Bruker Kappa APEXII diffractometer	2741 independent reflections
Graphite monochromator	1948 reflections with *I* > 2σ(*I*)
Detector resolution: 8.3333 pixels mm^-1^	*R*_int_ = 0.067
single crystal scans	θ_max_ = 25.4°, θ_min_ = 2.2°
Absorption correction: multi-scan (*SADABS*; Bruker, 2009)	*h* = −10→10
*T*_min_ = 0.77, *T*_max_ = 0.85	*k* = −15→15
19224 measured reflections	*l* = −15→16

### Refinement

**Table:**

Refinement on *F*^2^	Primary atom site location: structure-invariant direct methods
Least-squares matrix: full	Secondary atom site location: difference Fourier map
*R*[*F*^2^ > 2σ(*F*^2^)] = 0.041	Hydrogen site location: inferred from neighbouring sites
*wR*(*F*^2^) = 0.097	H atoms treated by a mixture of independent and constrained refinement
*S* = 1.00	*w* = 1/[σ^2^(*F*_o_^2^) + (0.0416*P*)^2^ + 2.7231*P*] where *P* = (*F*_o_^2^ + 2*F*_c_^2^)/3
2741 reflections	(Δ/σ)_max_ < 0.001
185 parameters	Δρ_max_ = 0.95 e Å^−^^3^
3 restraints	Δρ_min_ = −0.86 e Å^−^^3^

### Special details

**Table:**

Geometry. All e.s.d.'s (except the e.s.d. in the dihedral angle between two l.s. planes) are estimated using the full covariance matrix. The cell e.s.d.'s are taken into account individually in the estimation of e.s.d.'s in distances, angles and torsion angles; correlations between e.s.d.'s in cell parameters are only used when they are defined by crystal symmetry. An approximate (isotropic) treatment of cell e.s.d.'s is used for estimating e.s.d.'s involving l.s. planes.
Refinement. Refinement of *F*^2^ against ALL reflections. The weighted *R*-factor *wR* and goodness of fit *S* are based on *F*^2^, conventional *R*-factors *R* are based on *F*, with *F* set to zero for negative *F*^2^. The threshold expression of *F*^2^ > σ(*F*^2^) is used only for calculating *R*-factors(gt) *etc*. and is not relevant to the choice of reflections for refinement. *R*-factors based on *F*^2^ are statistically about twice as large as those based on *F*, and *R*- factors based on ALL data will be even larger.

### Fractional atomic coordinates and isotropic or equivalent isotropic displacement parameters (Å^2^)

**Table:**

	*x*	*y*	*z*	*U*_iso_*/*U*_eq_	
I1	0.01318 (5)	0.80017 (3)	0.33435 (3)	0.05632 (18)	
Ni1	0	0.5	1.0	0.0183 (2)	
C1	0.1600 (6)	0.4962 (4)	0.8111 (3)	0.0278 (11)	
H1	0.2538	0.4945	0.8623	0.033*	
C2	0.1749 (6)	0.4983 (4)	0.7105 (3)	0.0304 (12)	
H2	0.2763	0.5	0.6934	0.036*	
C3	0.0387 (6)	0.4978 (4)	0.6352 (3)	0.0290 (12)	
C4	−0.1044 (6)	0.4962 (4)	0.6634 (4)	0.0365 (13)	
H4	−0.1996	0.4952	0.6134	0.044*	
C5	−0.1092 (6)	0.4962 (4)	0.7655 (3)	0.0363 (13)	
H5	−0.2096	0.496	0.784	0.044*	
C6	0.1181 (6)	0.7073 (4)	0.9562 (4)	0.0320 (12)	
H6	0.1913	0.668	0.9286	0.038*	
C7	0.1276 (7)	0.8108 (4)	0.9503 (4)	0.0416 (14)	
H7	0.2052	0.842	0.9196	0.05*	
C8	0.0228 (7)	0.8671 (4)	0.9897 (4)	0.0402 (15)	
C9	−0.0855 (7)	0.8207 (4)	1.0356 (4)	0.0444 (15)	
H9	−0.1575	0.8591	1.0651	0.053*	
C10	−0.0877 (7)	0.7150 (4)	1.0381 (4)	0.0388 (14)	
H10	−0.1634	0.6824	1.0694	0.047*	
C11	0.8888 (11)	0.7821 (7)	0.7076 (6)	0.095 (3)	
H11A	0.9057	0.8424	0.7508	0.142*	
H11B	0.9055	0.7217	0.7504	0.142*	
H11C	0.7804	0.7823	0.6675	0.142*	
C12	1.0001 (11)	0.7823 (7)	0.6391 (7)	0.093 (3)	
H12A	1.1095	0.7802	0.6796	0.112*	
H12B	0.9832	0.7214	0.5957	0.112*	
N1	0.0212 (5)	0.4964 (3)	0.8404 (3)	0.0226 (9)	
N2	0.0617 (5)	0.4987 (3)	0.5323 (3)	0.0345 (10)	
N3	0.0113 (5)	0.6588 (3)	0.9985 (3)	0.0240 (9)	
N4	0.0347 (6)	0.9783 (4)	0.9742 (4)	0.0490 (13)	
O1	0.2450 (4)	0.4913 (3)	1.0428 (2)	0.0263 (8)	
H1A	0.305 (5)	0.539 (3)	1.062 (4)	0.039*	
H1B	0.293 (6)	0.442 (3)	1.074 (3)	0.039*	
O2	0.9803 (5)	0.8708 (3)	0.5759 (3)	0.0568 (12)	
H2A	0.9192	0.8576	0.5203	0.085*	

### Atomic displacement parameters (Å^2^)

**Table:**

	*U*^11^	*U*^22^	*U*^33^	*U*^12^	*U*^13^	*U*^23^
I1	0.0540 (3)	0.0502 (3)	0.0633 (3)	−0.0059 (2)	0.0085 (2)	−0.0198 (2)
Ni1	0.0260 (5)	0.0134 (4)	0.0159 (4)	0.0006 (4)	0.0054 (3)	0.0000 (3)
C1	0.025 (3)	0.038 (3)	0.019 (2)	0.001 (2)	0.001 (2)	0.000 (2)
C2	0.028 (3)	0.041 (3)	0.024 (2)	−0.001 (2)	0.011 (2)	−0.001 (2)
C3	0.040 (3)	0.030 (3)	0.017 (2)	0.002 (2)	0.005 (2)	−0.002 (2)
C4	0.024 (3)	0.061 (4)	0.025 (3)	0.002 (3)	0.005 (2)	0.003 (3)
C5	0.030 (3)	0.057 (4)	0.024 (3)	0.000 (3)	0.010 (2)	−0.001 (2)
C6	0.033 (3)	0.024 (3)	0.038 (3)	0.001 (2)	0.007 (2)	0.003 (2)
C7	0.041 (3)	0.024 (3)	0.058 (4)	−0.001 (3)	0.006 (3)	0.009 (3)
C8	0.041 (4)	0.016 (3)	0.055 (3)	−0.009 (3)	−0.010 (3)	0.001 (2)
C9	0.054 (4)	0.027 (3)	0.052 (4)	0.013 (3)	0.008 (3)	−0.011 (3)
C10	0.055 (4)	0.025 (3)	0.038 (3)	0.000 (3)	0.016 (3)	−0.001 (2)
C11	0.103 (7)	0.109 (7)	0.068 (5)	−0.001 (6)	0.008 (5)	0.033 (5)
C12	0.085 (6)	0.091 (7)	0.106 (7)	0.004 (5)	0.025 (5)	0.033 (5)
N1	0.028 (2)	0.019 (2)	0.0208 (19)	−0.0026 (18)	0.0066 (17)	0.0008 (17)
N2	0.038 (3)	0.050 (3)	0.018 (2)	0.000 (2)	0.0098 (16)	0.000 (2)
N3	0.032 (2)	0.019 (2)	0.0202 (19)	−0.0020 (19)	0.0026 (17)	−0.0018 (16)
N4	0.044 (3)	0.050 (3)	0.056 (3)	0.002 (3)	0.017 (2)	−0.008 (2)
O1	0.027 (2)	0.024 (2)	0.0267 (17)	0.0012 (15)	0.0034 (15)	0.0030 (14)
O2	0.056 (3)	0.054 (3)	0.060 (3)	0.006 (2)	0.011 (2)	0.008 (2)

### Geometric parameters (Å, º)

**Table:**

Ni1—O1^i^	2.080 (3)	C7—C8	1.360 (8)
Ni1—O1	2.080 (3)	C7—H7	0.95
Ni1—N3	2.109 (4)	C8—C9	1.366 (8)
Ni1—N3^i^	2.109 (4)	C8—N4	1.496 (7)
Ni1—N1	2.186 (3)	C9—C10	1.403 (8)
Ni1—N1^i^	2.186 (3)	C9—H9	0.95
C1—N1	1.336 (6)	C10—N3	1.324 (7)
C1—C2	1.381 (6)	C10—H10	0.95
C1—H1	0.95	C11—C12	1.458 (12)
C2—C3	1.386 (7)	C11—H11A	0.98
C2—H2	0.95	C11—H11B	0.98
C3—C4	1.365 (7)	C11—H11C	0.98
C3—N2	1.435 (6)	C12—O2	1.437 (9)
C4—C5	1.378 (7)	C12—H12A	0.99
C4—H4	0.95	C12—H12B	0.99
C5—N1	1.348 (6)	N2—N2^ii^	1.229 (8)
C5—H5	0.95	N4—N4^iii^	1.156 (9)
C6—N3	1.342 (7)	O1—H1A	0.82 (2)
C6—C7	1.378 (7)	O1—H1B	0.833 (19)
C6—H6	0.95	O2—H2A	0.84
			
O1^i^—Ni1—O1	180.00 (19)	C6—C7—H7	120.9
O1^i^—Ni1—N3	89.33 (15)	C7—C8—C9	119.9 (5)
O1—Ni1—N3	90.68 (15)	C7—C8—N4	114.7 (5)
O1^i^—Ni1—N3^i^	90.67 (15)	C9—C8—N4	125.3 (6)
O1—Ni1—N3^i^	89.33 (15)	C8—C9—C10	118.3 (6)
N3—Ni1—N3^i^	180.0 (2)	C8—C9—H9	120.9
O1^i^—Ni1—N1	90.79 (13)	C10—C9—H9	120.9
O1—Ni1—N1	89.21 (13)	N3—C10—C9	122.7 (5)
N3—Ni1—N1	89.97 (15)	N3—C10—H10	118.7
N3^i^—Ni1—N1	90.03 (15)	C9—C10—H10	118.7
O1^i^—Ni1—N1^i^	89.21 (13)	C12—C11—H11A	109.5
O1—Ni1—N1^i^	90.79 (13)	C12—C11—H11B	109.5
N3—Ni1—N1^i^	90.03 (15)	H11A—C11—H11B	109.5
N3^i^—Ni1—N1^i^	89.97 (15)	C12—C11—H11C	109.5
N1—Ni1—N1^i^	180.0 (2)	H11A—C11—H11C	109.5
N1—C1—C2	123.7 (4)	H11B—C11—H11C	109.5
N1—C1—H1	118.1	O2—C12—C11	111.0 (7)
C2—C1—H1	118.1	O2—C12—H12A	109.4
C1—C2—C3	118.6 (5)	C11—C12—H12A	109.4
C1—C2—H2	120.7	O2—C12—H12B	109.4
C3—C2—H2	120.7	C11—C12—H12B	109.4
C4—C3—C2	118.7 (4)	H12A—C12—H12B	108.0
C4—C3—N2	125.3 (4)	C1—N1—C5	116.3 (4)
C2—C3—N2	116.0 (5)	C1—N1—Ni1	123.2 (3)
C3—C4—C5	119.2 (5)	C5—N1—Ni1	120.5 (3)
C3—C4—H4	120.4	N2^ii^—N2—C3	114.1 (5)
C5—C4—H4	120.4	C10—N3—C6	117.1 (4)
N1—C5—C4	123.5 (5)	C10—N3—Ni1	121.6 (4)
N1—C5—H5	118.2	C6—N3—Ni1	121.3 (3)
C4—C5—H5	118.2	N4^iii^—N4—C8	110.5 (7)
N3—C6—C7	123.7 (5)	Ni1—O1—H1A	126 (4)
N3—C6—H6	118.1	Ni1—O1—H1B	124 (4)
C7—C6—H6	118.1	H1A—O1—H1B	103 (5)
C8—C7—C6	118.2 (5)	C12—O2—H2A	109.5
C8—C7—H7	120.9		

Symmetry codes: (i) −*x*, −*y*+1, −*z*+2; (ii) −*x*, −*y*+1, −*z*+1; (iii) −*x*, −*y*+2, −*z*+2.

### Hydrogen-bond geometry (Å, º)

**Table:**

*D*—H···*A*	*D*—H	H···*A*	*D*···*A*	*D*—H···*A*
O1—H1*A*···O2^iv^	0.83 (4)	1.91 (4)	2.703 (6)	161 (5)

Symmetry code: (iv) *x*−1/2, −*y*+3/2, *z*+1/2.

## References

[bb1] Aijaz, A., Sañudo, E. C. & Bharadwaj, P. K. (2011). *Cryst. Growth Des.* **11**, 1122–1134.

[bb2] Bruker (2009). *APEX2*, *SADABS* and *SAINT* Bruker AXS Inc., Madison, Wisconsin, USA.

[bb3] Carlucci, L., Ciani, G., Proserpio, D. M. & Rizzato, S. (2003). *CrystEngComm*, **5**, 190–199.

[bb4] Li, S.-L., Lan, Y.-Q., Ma, J.-F., Yang, J., Wang, X.-H. & Su, Z.-M. (2007). *Inorg. Chem.* **46**, 8283–8290. 10.1021/ic700913m 17824699

[bb5] Noro, S.-I., Kitagawa, S., Nakamura, T. & Wada, T. (2005). *Inorg. Chem.* **44**, 3960–3971. 10.1021/ic048371u 15907124

[bb6] Noro, S.-I., Kitaura, R., Kitagawa, S., Akutagawa, T. & Nakamura, T. (2006). *Inorg. Chem.* **45**, 8990–8997. 10.1021/ic061052d 17054359

[bb7] Pan, F., Wu, J., Hou, H. & Fan, Y. (2010). *Cryst. Growth Des.* **10**, 3835–3837.

[bb8] Sheldrick, G. M. (2008). *Acta Cryst.* A**64**, 112–122. 10.1107/S0108767307043930 18156677

