# Supplementary material for: Crystal structure of poly[[trans-di­aqua­bis­[μ2-trans-4,4′-(diazenedi­yl)dipyridine]­nickel(II)] diiodide ethanol disolvate]
Source: Acta Crystallogr Sect E Struct Rep Online. 2014 Aug 1;70(Pt 9):m314–5. doi: 10.1107/S1600536814016158 (PMC4186204; doi:10.1107/S1600536814016158)
Supplement: Supplementary file 4 [file e-70-0m314-Isup4.docx]

***Catena*-{*trans*-[diaquabis(µ_2_-*trans*-4,4’-azopyridine)nickel(II)]} iodide−ethanol (1/2)**

Miguel Cortijo, Santiago Herrero, and Josefina Perles

*Departamento de Química Inorgánica, Facultad de Ciencias Químicas, Universidad Complutense de Madrid, Ciudad Universitaria, E-28040 Madrid, Spain.*

In the title compound, [Ni(OH_2_)_2_(*t*-apy)_2_]I_2_·2EtOH (*t*-apy = *trans*-4,4’-azopyridine), the coordination environment of the nickel atoms is pseudo-octahedral (NiN_4_O_2_) [Ni−N = 2.109(4) Å, and 2.186(3), Ni−O = 2.080(3) Å]. The metal atoms are coordinated to two water molecules in a *trans* disposition and four 4,4'-azopyridine ligands bridging Ni atoms along the *b* and *c* directions giving rise to a two dimensional arrangement. The complex crystallizes together with two iodine anions and two ethanol molecules per nickel atom situated in the voids of the net. Both species are connected to the water molecules from neighbour layers along the *a* axis through hydrogen bonds.

**Related literature**

For related 2D structures see: Carlucci *et al.* (2003); Noro *et al.* (2005 and 2006); Li *et al.* (2007); Pan *et al.* (2010); and Aijaz *et al.* (2011).





**Experimental**

*Crystal data*

| [Ni(OH_2_)_2_(*t*-apy)_2_]I_2_·2EtOH | *V* = 1504.6(3) Å^3^ |
| --- | --- |
| *M_r_* = 809.09 | *Z* = 2 |
| Monoclinic, *P* 2_1_/*n* | Mo *K*α radiation |
| *a* = 8.6367(11) Å | *µ* = 2.740 mm^−1^ |
| *b* = 13.2598(16) Å | *T* = 100 K |
| *c* = 13.4188(14) Å | 0.06 x 0.08 x 0.12 mm |
| *β* = 101.737(3)° |  |

*Data collection*

| Kappa Apex II Bruker diffractometer | 19224 measured reflections |
| --- | --- |
| Absorption correction: multi-scan method | 2741 independent reflections |
| SADABS (Bruker, 2005) | 1948 reflections with *I* ˃2σ(*I*) |
| T*_min_* = 0.77 T*_max_* = 0.85 | R*_int_* = 0.0672 |

*Refinement*

| *R*[*F^2^*˃2σ(*F^2^*)] = 0.0407 | 185 parameters |
| --- | --- |
| *wR*(*F^2^*) = 0.0850 | H-atoms treated by constrained refinement |
| *S* = 1.000 | *∆ρ*_max_ = 0.946 eÅ^−3^ |
| 2741 reflections | *∆ρ*_min_ −0.855 eÅ^−3^ |

Data collection: *Bruker SHELXTL Software Package* (Sheldrick, 2008); cell refinement: *SAINT V7.68A* (Bruker AXS, 2009)*;* data reduction: *SAINT V7.68A* (Bruker AXS, 2009); program(s) used to solve structure: *SHELXS-97* (Sheldrick, 2008); program(s) used to refine structure: *SHELXS-97* (Sheldrick, 2008). Diffraction data were collected at the SCXRD laboratory from the Servicio Intedepartamental de Investigación (UAM).

Financial support received from the Spanish Ministerio de Economía y Competitividad (CTQ2011-23066) and Comunidad de Madrid (S2009/MAT-1467) is gratefully acknowledged.

**References**

Aijaz, A., Sañudo, E. C., Bharadwaj, P. K. (2011) *Cryst. Growth Des.* **11**, 1122–1134.

SADABS and SAINT. Bruker AXS Inc., Madison, Wisconsin, USA (2009).

[Carlucci](http://pubs.rsc.org/en/results?searchtext=Author%3ALucia%20Carlucci), L., [Ciani](http://pubs.rsc.org/en/results?searchtext=Author%3AGianfranco%20Ciani), G., [Proserpio](http://pubs.rsc.org/en/results?searchtext=Author%3ADavide%20M.%20Proserpio), D. M., [Rizzato](http://pubs.rsc.org/en/results?searchtext=Author%3ASilvia%20Rizzato), S. (2003) *CrystEngComm* **5**, 190−199.

Li, S.-L., Lan, Y.-Q., Ma, J.-F., Yang, J., Wang, X.-H., Su, Z.-M. (2007) *Inorg. Chem.* **46**, 8283−8290.

Noro, S.-I., Kitagawa, S., Nakamura, T., Wada, T. (2005) *Inorg. Chem.* **44**, 3960−3971.

Noro, S.-I., Kitaura, R., Kitagawa, S., Akutagawa, T., Nakamura, T. (2006) *Inorg. Chem.* **45**, 8990−8997.

Pan, F., Wu, J., Hou, H., Fan, Y. (2010) *Cryst. Growth Des.* **10**, 3835–3837.

Sheldrick, G. M. (2008). *Acta Cryst.* A**64**, 112-122.

**Supplementary materials**

**1. Comment**

A similar laminar structure was found for the compound [Ni(NCS)_2_(*t*-apy)_2_]·3toluene (Noro, 2006) although in this latter case there is no one-dimensional H-bond chain.

**2. Experimental**

Nickel(II) iodide (0.30 g, 1.0 mmol), *trans*-4,4'-azopyridine (0.18 g, 1.0 mmol), ethanol (9 mL), and water (3 mL) were placed into an 85 mL Teflon vessel with a magnetic stirrer. The vessel was sealed with a lid equipped with a temperature sensor and placed in a ETHOS ONE microwave oven. The reaction mixture was heated for 3 hours at 120 ºC and left to cool afterwards. Slow interdiffusion of diethyl ether in the obtained solution gave rise to red crystals suitable for single-crystal X-ray diffraction after a few days.


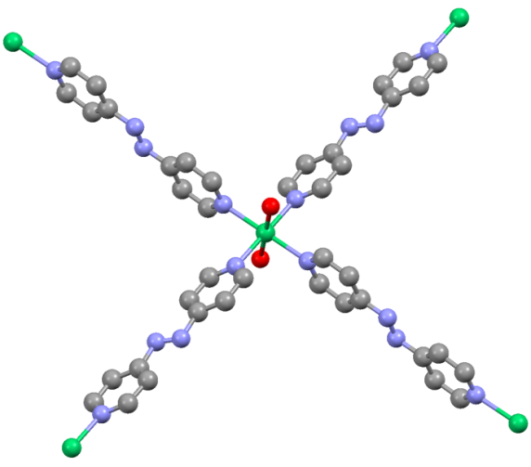


Figure 1. Simplified drawing of the coordination environment of the nickel atom. Hydrogen atoms have been omitted for clarity.


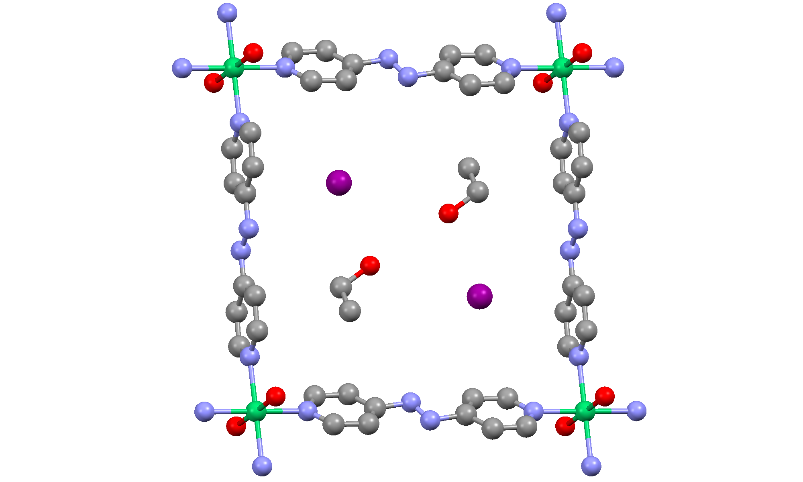


Figure 2. Simplified drawing of a *bc* layer. Hydrogen atoms have been omitted for clarity.


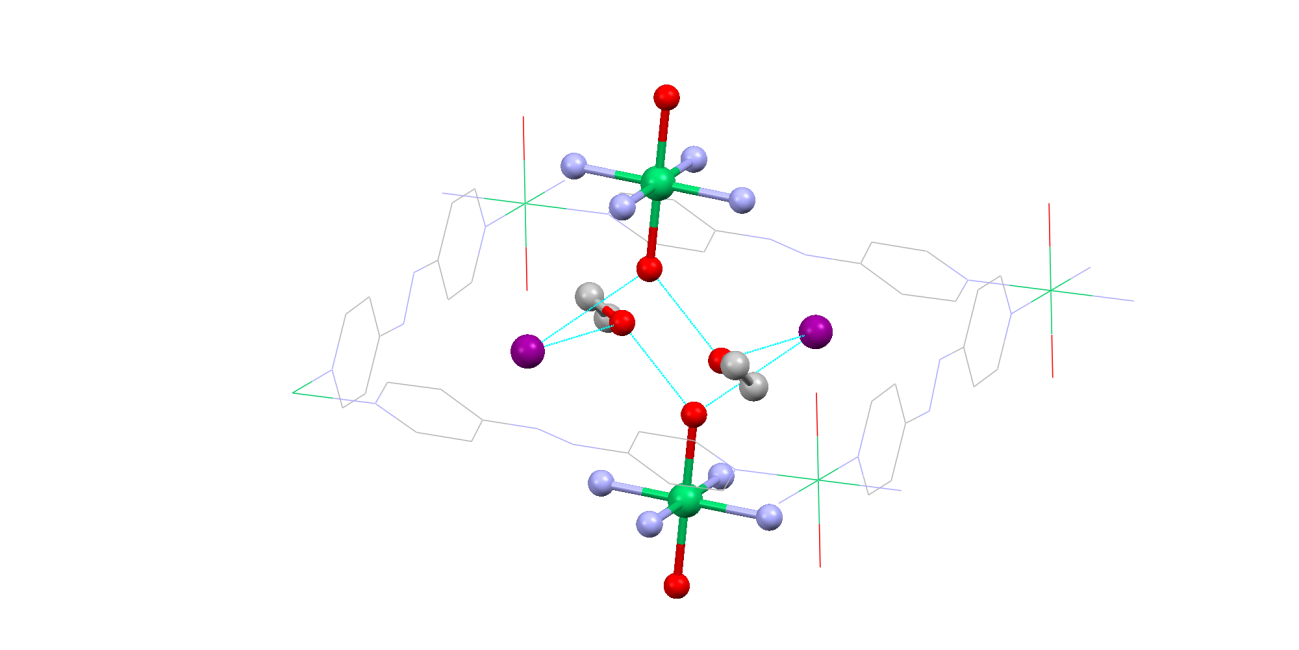


Figure 3. Hydrogen bonds between the coordinated water molecules and the ethanol and iodide fragments located in the interstices.

A clear orange-red prismatic-like specimen of C_24_H_32_I_2_N_8_NiO_4_, approximate dimensions 0.06 mm x 0.08 mm x 0.12 mm, was used for the X-ray crystallographic analysis. The X-ray intensity data were measured.

**Data collection details**

| **Axis** | **dx/mm** | **2θ/°** | **ω/°** | **φ/°** | **χ/°** | **Width/°** | **Frames** | **Time/s** | **λ/**Å | **V/kV** | **Current/mA** | **T** |
| --- | --- | --- | --- | --- | --- | --- | --- | --- | --- | --- | --- | --- |
| Phi | 35.048 | 13.00 | 13.95 | -39.72 | -74.52 | 1.00 | 369 | 90.00 | 0.71073 | 50 | 30.0 | n/a |
| Phi | 35.048 | 20.50 | 12.48 | -272.45 | 28.87 | 1.00 | 294 | 90.00 | 0.71073 | 50 | 30.0 | n/a |
| Omega | 35.048 | 20.50 | -37.87 | -65.82 | 79.36 | 1.00 | 48 | 90.00 | 0.71073 | 50 | 30.0 | n/a |
| Omega | 35.048 | -9.50 | -11.43 | -145.05 | -53.50 | 1.00 | 62 | 90.00 | 0.71073 | 50 | 30.0 | n/a |

A total of 773 frames were collected. The total exposure time was 19.32 hours. The frames were integrated with the Bruker SAINT software package using a narrow-frame algorithm. The integration of the data using a monoclinic unit cell yielded a total of 19224 reflections to a maximum θ angle of 25.35° (0.83 Å resolution), of which 2741 were independent (average redundancy 7.013, completeness = 99.4%, R_int_ = 6.72%, R_sig_ = 4.99%) and 1948 (71.07%) were greater than 2σ(F^2^). The final cell constants of *a* = 8.6367(11) Å, *b* = 13.2598(16) Å, *c* = 13.4188(14) Å, β = 101.737(3), volume = 1504.6(3) Å^3^, are based upon the refinement of the XYZ-centroids of 3456 reflections above 20 σ(I) with 5.713° < 2θ < 43.17°. Data were corrected for absorption effects using the multi-scan method (SADABS). The ratio of minimum to maximum apparent transmission was 0.897. The calculated minimum and maximum transmission coefficients (based on crystal size) are 0.7345 and 0.8529.

The structure was solved and refined using the Bruker SHELXTL Software Package, using the space group *P*2_1_/*n*, with Z = 2 for the formula unit,C_24_H_32_I_2_N_8_NiO_4_. The final anisotropic full-matrix least-squares refinement on F^2^ with 185 variables converged at R1 = 4.07%, for the observed data and wR2 = 9.72% for all data. The goodness-of-fit was 1.000. The largest peak in the final difference electron density synthesis was 0.946 e^-^/Å^3^ and the largest hole was -0.855 e^-^/Å^3^ with an RMS deviation of 0.106 e^-^/Å^3^. On the basis of the final model, the calculated density was 1.786 g/cm^3^ and F(000), 796 e^-^.

**Sample and crystal data**

| **Chemical formula** | C_24_H_32_I_2_N_8_NiO_4_ | |
| --- | --- | --- |
| **Formula weight** | 809.09 | |
| **Temperature** | 100(2) K | |
| **Wavelength** | 0.71073 Å | |
| **Crystal size** | 0.06 x 0.08 x 0.12 mm | |
| **Crystal habit** | clear orange-red prismatic | |
| **Crystal system** | monoclinic | |
| **Space group** | *P*2_1_/*n* | |
| **Unit cell dimensions** | *a* = 8.6367(11) Å  *b* = 13.2598(16) Å  *c* = 13.4188(14) Å | α = 90°  β = 101.737(3)°  γ = 90° |
| **Volume** | 1504.6(3) Å^3^ |  |
| **Z** | 2 | |
| **Density (calculated)** | 1.786 Mg/cm^3^ | |
| **Absorption coefficient** | 2.740 mm^-1^ | |
| **F(000)** | 796 | |

**Data collection and structure refinement**

| **Theta range for data collection** | 2.18 to 25.35° | |
| --- | --- | --- |
| **Index ranges** | -10<=h<=10, -15<=k<=15, -15<=l<=16 | |
| **Reflections collected** | 19224 | |
| **Independent reflections** | 2741 [R(int) = 0.0672] | |
| **Coverage of independent reflections** | 99.4% | |
| **Absorption correction** | multi-scan | |
| **Max. and min. transmission** | 0.8529 and 0.7345 | |
| **Structure solution technique** | direct methods | |
| **Structure solution program** | SHELXS-97 (Sheldrick, 2008) | |
| **Refinement method** | Full-matrix least-squares on F^2^ | |
| **Refinement program** | SHELXL-97 (Sheldrick, 2008) | |
| **Function minimized** | Σ w(F_o_^2^ - F_c_^2^)^2^ | |
| **Data / restraints / parameters** | 2741 / 3 / 185 | |
| **Goodness-of-fit on F^2^** | 1.000 | |
| **Final R indices** | 1948 data; I>2σ(I); | R1 = 0.0407, wR2 = 0.0850 |
|  | all data; | R1 = 0.0736, wR2 = 0.0972 |
| **Weighting scheme** | w=1/[σ^2^(F_o_^2^)+(0.0416P)^2^+2.7231P]  where P=(F_o_^2^+2F_c_^2^)/3 | |
| **Largest diff. peak and hole** | 0.946 and -0.855 eÅ^-3^ | |
| **R.M.S. deviation from mean** | 0.106 eÅ^-3^ | |

**Atomic coordinates and equivalent isotropic atomic displacement parameters (Å^2^)**

| U(eq) is defined as one third of the trace of the orthogonalized U_ij_ tensor. |
| --- |
|  |

|  | **x/a** | **y/b** | **z/c** | **U(eq)** |
| --- | --- | --- | --- | --- |
| I1 | 0.01318(5) | 0.80017(3) | 0.33435(3) | 0.05632(18) |
| Ni1 | 0.0 | 0.5 | 0.0 | 0.0183(2) |
| C1 | 0.1600(6) | 0.4962(4) | 0.8111(3) | 0.0278(11) |
| C2 | 0.1749(6) | 0.4983(4) | 0.7105(3) | 0.0304(12) |
| C3 | 0.0387(6) | 0.4978(4) | 0.6352(3) | 0.0290(12) |
| C4 | 0.8956(6) | 0.4962(4) | 0.6634(4) | 0.0365(13) |
| C5 | 0.8908(6) | 0.4962(4) | 0.7655(3) | 0.0363(13) |
| C6 | 0.1181(6) | 0.7073(4) | 0.9562(4) | 0.0320(12) |
| C7 | 0.1276(7) | 0.8108(4) | 0.9503(4) | 0.0416(14) |
| C8 | 0.0228(7) | 0.8671(4) | 0.9897(4) | 0.0402(15) |
| C9 | 0.9145(7) | 0.8207(4) | 0.0356(4) | 0.0444(15) |
| C10 | 0.9123(7) | 0.7150(4) | 0.0381(4) | 0.0388(14) |
| C11 | 0.8888(11) | 0.7821(7) | 0.7076(6) | 0.095(3) |
| C12 | 0.0001(11) | 0.7823(7) | 0.6391(7) | 0.093(3) |
| N1 | 0.0212(5) | 0.4964(3) | 0.8404(3) | 0.0226(9) |
| N2 | 0.0617(5) | 0.4987(3) | 0.5323(3) | 0.0345(10) |
| N3 | 0.0113(5) | 0.6588(3) | 0.9985(3) | 0.0240(9) |
| N4 | 0.0347(6) | 0.9783(4) | 0.9742(4) | 0.0490(13) |
| O1 | 0.2450(4) | 0.4913(3) | 0.0428(2) | 0.0263(8) |
| O2 | 0.9803(5) | 0.8708(3) | 0.5759(3) | 0.0568(12) |

**Bond lengths (Å)**

| Ni1-O1#2 | 2.080(3) | Ni1-O1 | 2.080(3) |
| --- | --- | --- | --- |
| Ni1-N3 | 2.109(4) | Ni1-N3#2 | 2.109(4) |
| Ni1-N1 | 2.186(3) | Ni1-N1#2 | 2.186(3) |
| C1-N1 | 1.336(6) | C1-C2 | 1.381(6) |
| C1-H1 | 0.95 | C2-C3 | 1.386(7) |
| C2-H2 | 0.95 | C3-C4 | 1.365(7) |
| C3-N2 | 1.435(6) | C4-C5 | 1.378(7) |
| C4-H4 | 0.95 | C5-N1 | 1.348(6) |
| C5-H5 | 0.95 | C6-N3 | 1.342(7) |
| C6-C7 | 1.378(7) | C6-H6 | 0.95 |
| C7-C8 | 1.360(8) | C7-H7 | 0.95 |
| C8-C9 | 1.366(8) | C8-N4 | 1.496(7) |
| C9-C10 | 1.403(8) | C9-H9 | 0.95 |
| C10-N3 | 1.324(7) | C10-H10 | 0.95 |
| C11-C12 | 1.458(12) | C11-H11A | 0.98 |
| C11-H11B | 0.98 | C11-H11C | 0.98 |
| C12-O2 | 1.437(9) | C12-H12A | 0.99 |
| C12-H12B | 0.99 | N2-N2#1 | 1.229(8) |
| N4-N4#3 | 1.156(9) | O1-H1A | 0.82(2) |
| O1-H1B | 0.833(19) | O2-H2A | 0.84 |

Symmetry transformations used to generate equivalent atoms:

| #1 | -x, -y+1, -z+1 |
| --- | --- |
| #2 | -x, -y+1, -z+2 |
| #3 | -x, -y+2, -z+2 |

**Bond angles (°)**

| O1#2-Ni1-O1 | 180.00(19) | O1#2-Ni1-N3 | 89.33(15) |
| --- | --- | --- | --- |
| O1-Ni1-N3 | 90.68(15) | O1#2-Ni1-N3#2 | 90.67(15) |
| O1-Ni1-N3#2 | 89.33(15) | N3-Ni1-N3#2 | 180.0(2) |
| O1#2-Ni1-N1 | 90.79(13) | O1-Ni1-N1 | 89.21(13) |
| N3-Ni1-N1 | 89.97(15) | N3#2-Ni1-N1 | 90.03(15) |
| O1#2-Ni1-N1#2 | 89.21(13) | O1-Ni1-N1#2 | 90.79(13) |
| N3-Ni1-N1#2 | 90.03(15) | N3#2-Ni1-N1#2 | 89.97(15) |
| N1-Ni1-N1#2 | 180.0(2) | N1-C1-C2 | 123.7(4) |
| N1-C1-H1 | 118.1 | C2-C1-H1 | 118.1 |
| C1-C2-C3 | 118.6(5) | C1-C2-H2 | 120.7 |
| C3-C2-H2 | 120.7 | C4-C3-C2 | 118.7(4) |
| C4-C3-N2 | 125.3(4) | C2-C3-N2 | 116.0(5) |
| C3-C4-C5 | 119.2(5) | C3-C4-H4 | 120.4 |
| C5-C4-H4 | 120.4 | N1-C5-C4 | 123.5(5) |
| N1-C5-H5 | 118.2 | C4-C5-H5 | 118.2 |
| N3-C6-C7 | 123.7(5) | N3-C6-H6 | 118.1 |
| C7-C6-H6 | 118.1 | C8-C7-C6 | 118.2(5) |
| C8-C7-H7 | 120.9 | C6-C7-H7 | 120.9 |
| C7-C8-C9 | 119.9(5) | C7-C8-N4 | 114.7(5) |
| C9-C8-N4 | 125.3(6) | C8-C9-C10 | 118.3(6) |
| C8-C9-H9 | 120.9 | C10-C9-H9 | 120.9 |
| N3-C10-C9 | 122.7(5) | N3-C10-H10 | 118.7 |
| C9-C10-H10 | 118.7 | C12-C11-H11A | 109.5 |
| C12-C11-H11B | 109.5 | H11A-C11-H11B | 109.5 |
| C12-C11-H11C | 109.5 | H11A-C11-H11C | 109.5 |
| H11B-C11-H11C | 109.5 | O2-C12-C11 | 111.0(7) |
| O2-C12-H12A | 109.4 | C11-C12-H12A | 109.4 |
| O2-C12-H12B | 109.4 | C11-C12-H12B | 109.4 |
| H12A-C12-H12B | 108.0 | C1-N1-C5 | 116.3(4) |
| C1-N1-Ni1 | 123.2(3) | C5-N1-Ni1 | 120.5(3) |
| N2#1-N2-C3 | 114.1(5) | C10-N3-C6 | 117.1(4) |
| C10-N3-Ni1 | 121.6(4) | C6-N3-Ni1 | 121.3(3) |
| N4#3-N4-C8 | 110.5(7) | Ni1-O1-H1A | 126.(4) |
| Ni1-O1-H1B | 124.(4) | H1A-O1-H1B | 103.(5) |
| C12-O2-H2A | 109.5 |  |  |

Symmetry transformations used to generate equivalent atoms:

| #1 | -x, -y+1, -z+1 |
| --- | --- |
| #2 | -x, -y+1, -z+2 |
| #3 | -x, -y+2, -z+2 |
|  |  |

**Anisotropic atomic displacement parameters (Å^2^)**

The anisotropic atomic displacement factor exponent takes the form: -2π^2^[ h^2^ a^*2^ U_11_ + ... + 2 h k a^*^ b^*^ U_12_ ]

|  | **U_11_** | **U_22_** | **U_33_** | **U_23_** | **U_13_** | **U_12_** |  |
| --- | --- | --- | --- | --- | --- | --- | --- |
| I1 | 0.0540(3) | 0.0502(3) | 0.0633(3) | -0.0198(2) | 0.0085(2) | -0.0059(2) |  |
| Ni1 | 0.0260(5) | 0.0134(4) | 0.0159(4) | 0.0000(3) | 0.0054(3) | 0.0006(4) |  |
| C1 | 0.025(3) | 0.038(3) | 0.019(2) | 0.000(2) | 0.001(2) | 0.001(2) |  |
| C2 | 0.028(3) | 0.041(3) | 0.024(2) | -0.001(2) | 0.011(2) | -0.001(2) |  |
| C3 | 0.040(3) | 0.030(3) | 0.017(2) | -0.002(2) | 0.005(2) | 0.002(2) |  |
| C4 | 0.024(3) | 0.061(4) | 0.025(3) | 0.003(3) | 0.005(2) | 0.002(3) |  |
| C5 | 0.030(3) | 0.057(4) | 0.024(3) | -0.001(2) | 0.010(2) | 0.000(3) |  |
| C6 | 0.033(3) | 0.024(3) | 0.038(3) | 0.003(2) | 0.007(2) | 0.001(2) |  |
| C7 | 0.041(3) | 0.024(3) | 0.058(4) | 0.009(3) | 0.006(3) | -0.001(3) |  |
| C8 | 0.041(4) | 0.016(3) | 0.055(3) | 0.001(2) | -0.010(3) | -0.009(3) |  |
| C9 | 0.054(4) | 0.027(3) | 0.052(4) | -0.011(3) | 0.008(3) | 0.013(3) |  |
| C10 | 0.055(4) | 0.025(3) | 0.038(3) | -0.001(2) | 0.016(3) | 0.000(3) |  |
| C11 | 0.103(7) | 0.109(7) | 0.068(5) | 0.033(5) | 0.008(5) | -0.001(6) |  |
| C12 | 0.085(6) | 0.091(7) | 0.106(7) | 0.033(5) | 0.025(5) | 0.004(5) |  |
| N1 | 0.028(2) | 0.019(2) | 0.0208(19) | 0.0008(17) | 0.0066(17) | -0.0026(18) |  |
| N2 | 0.038(3) | 0.050(3) | 0.018(2) | 0.000(2) | 0.0098(16) | 0.000(2) |  |
| N3 | 0.032(2) | 0.019(2) | 0.0202(19) | -0.0018(16) | 0.0026(17) | -0.0020(19) |  |
| N4 | 0.044(3) | 0.050(3) | 0.056(3) | -0.008(2) | 0.017(2) | 0.002(3) |  |
| O1 | 0.027(2) | 0.024(2) | 0.0267(17) | 0.0030(14) | 0.0034(15) | 0.0012(15) |  |
| O2 | 0.056(3) | 0.054(3) | 0.060(3) | 0.008(2) | 0.011(2) | 0.006(2) |  |

**Hydrogen atomic coordinates and isotropic atomic displacement parameters (Å^2^)**

|  | **x/a** | **y/b** | **z/c** | **U(eq)** |
| --- | --- | --- | --- | --- |
| H1 | 0.2538 | 0.4945 | 0.8623 | 0.033 |
| H2 | 0.2763 | 0.5000 | 0.6934 | 0.036 |
| H4 | -0.1996 | 0.4952 | 0.6134 | 0.044 |
| H5 | -0.2096 | 0.4960 | 0.7840 | 0.044 |
| H6 | 0.1913 | 0.6680 | 0.9286 | 0.038 |
| H7 | 0.2052 | 0.8420 | 0.9196 | 0.05 |
| H9 | -0.1575 | 0.8591 | 1.0651 | 0.053 |
| H10 | -0.1634 | 0.6824 | 1.0694 | 0.047 |
| H11A | 0.9057 | 0.8424 | 0.7508 | 0.142 |
| H11B | 0.9055 | 0.7217 | 0.7504 | 0.142 |
| H11C | 0.7804 | 0.7823 | 0.6675 | 0.142 |
| H12A | 1.1095 | 0.7802 | 0.6796 | 0.112 |
| H12B | 0.9832 | 0.7214 | 0.5957 | 0.112 |
| H1A | 0.305(5) | 0.539(3) | 1.062(4) | 0.039 |
| H1B | 0.293(6) | 0.442(3) | 1.074(3) | 0.039 |
| H2A | 0.9192 | 0.8576 | 0.5203 | 0.085 |
