# Supplementary figures and images for: Crystal structure of poly[[trans-di­aqua­bis­[μ2-trans-4,4′-(diazenedi­yl)dipyridine]­nickel(II)] diiodide ethanol disolvate]
Source: Acta Crystallogr Sect E Struct Rep Online. 2014 Aug 1;70(Pt 9):m314–5. doi: 10.1107/S1600536814016158 (PMC4186204; doi:10.1107/S1600536814016158)

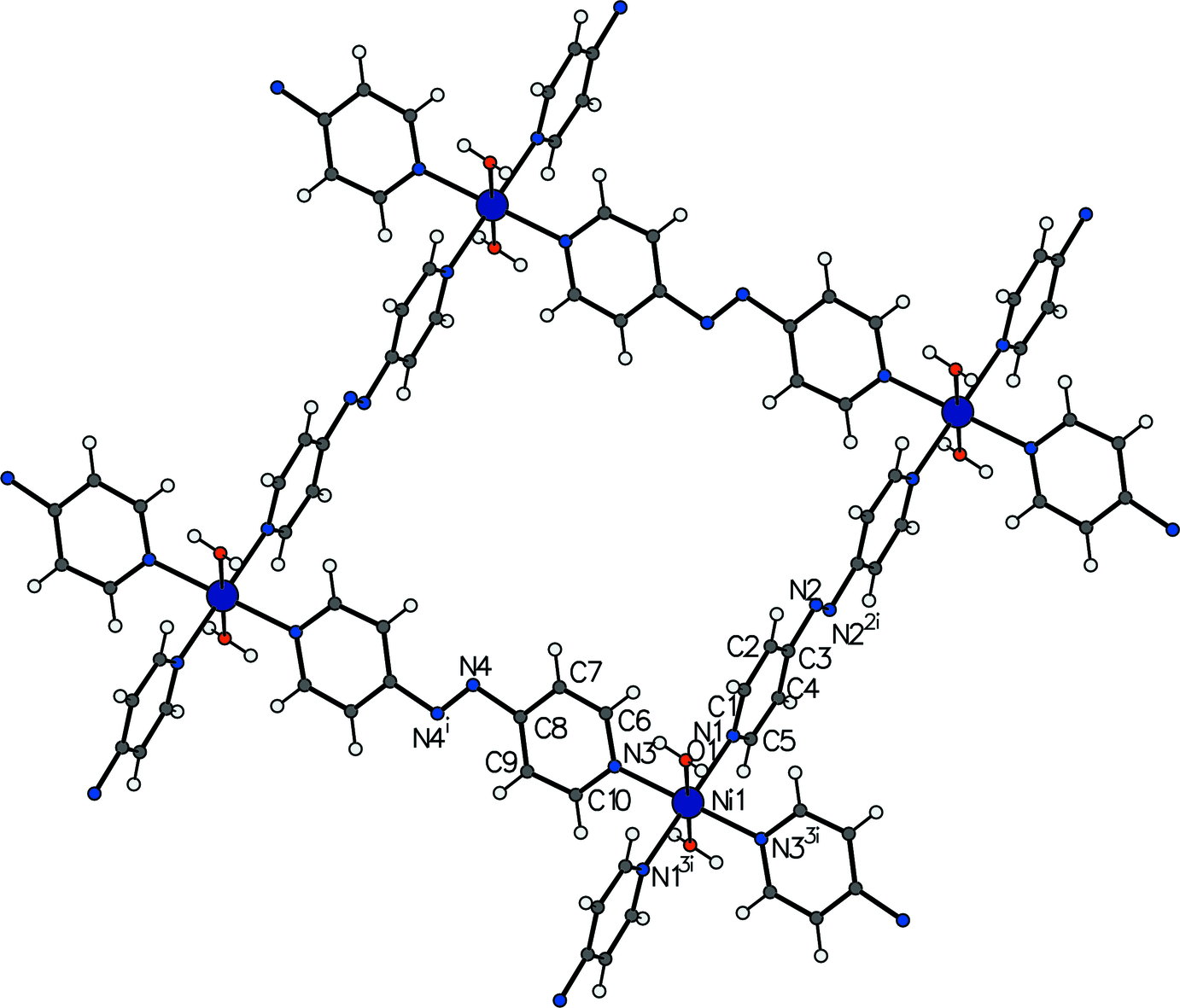

Supplement: Supplementary file 5 [file e-70-0m314-fig1.tif]

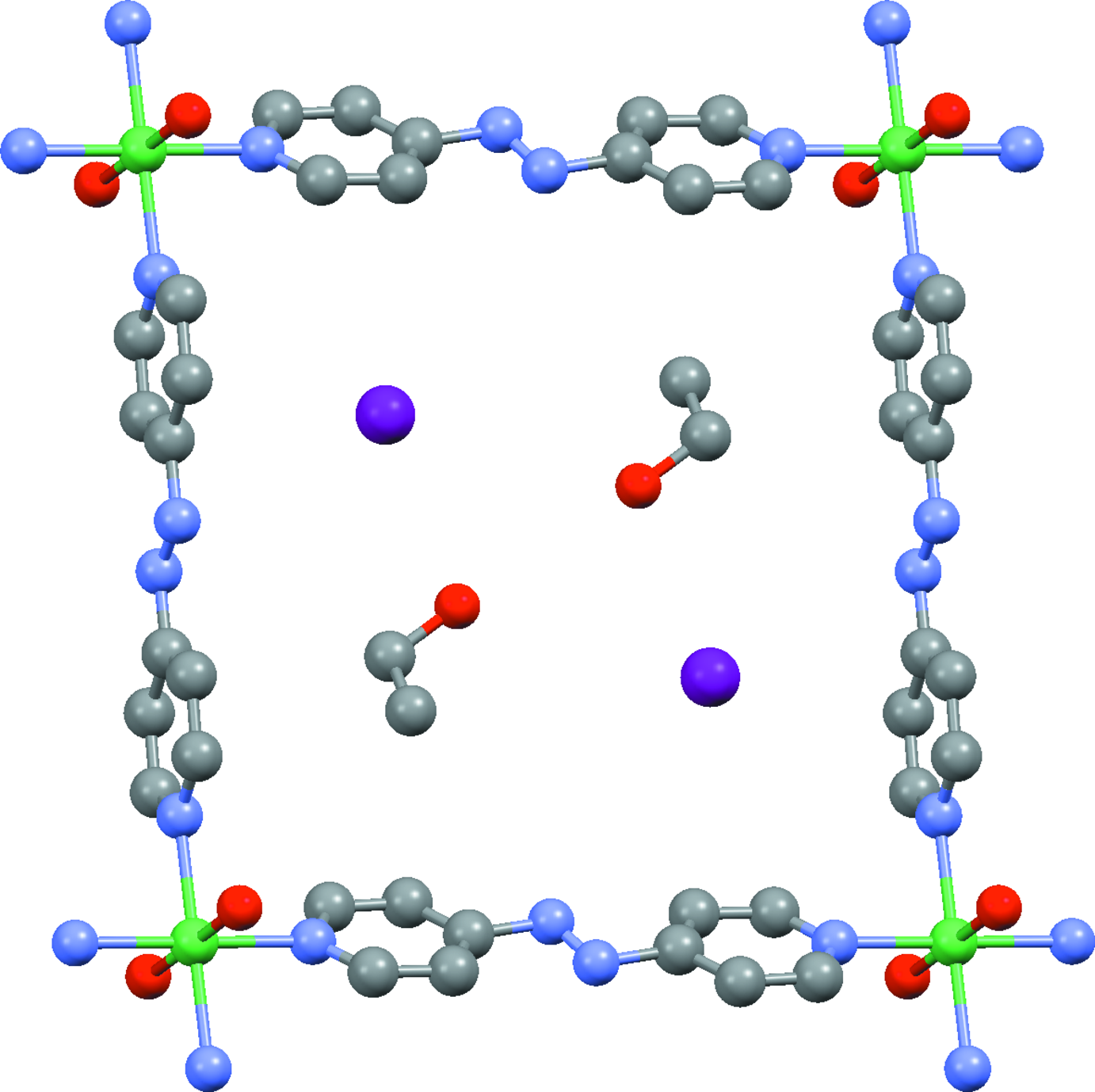

Supplement: Supplementary file 6 [file e-70-0m314-fig2.tif]
